# Bioassay-guided isolation, identification of compounds from *Origanum rotundifolium* and investigation of their antiproliferative and antioxidant activities

**DOI:** 10.1080/13880209.2017.1310906

**Published:** 2017-04-21

**Authors:** Ramazan Erenler, Bilal Meral, Ozkan Sen, Mahfuz Elmastas, Ali Aydin, Ozgur Eminagaoglu, Gulacti Topcu

**Affiliations:** a Department of Chemistry, Faculty of Art and Science, Gaziosmanpasa University, Tokat, Turkey;; b Department of Biology, Faculty of Art and Science, Gaziosmanpasa University, Tokat, Turkey;; c Department of Forest Engineering, Faculty of Foresty, Artvin Coruh University, Artvin, Turkey;; d Department of Pharmacognosy and Phytochemistry, Faculty of Pharmacy, Bezmialem Vakif University, Istanbul, Turkey

**Keywords:** Secondary metabolites, isolation, chromatography, spectroscopy

## Abstract

**Context:** *Origanum* (Lamiaceae) has been used in food and pharmaceutical industries.

**Objective:** Isolation and identification of bioactive compounds from *Origanum rotundifolium* Boiss. and investigation of their antiproliferative and antioxidant activities.

**Materials and methods:** The aerial part of *O. rotundifolium* was dried and powdered (1.0 kg ±2.0 g) then extracted with hexane, ethyl acetate, methanol and water. Solvent (3 × 1 L) was used for each extraction for a week at room temperature. The aqueous extract was partitioned with ethyl acetate (3 × 1 L) to yield the water/EtOAc extract subjected to chromatography to isolate the active compounds. The structures of isolated compounds were elucidated by 1 D, 2 D NMR and LC-TOF/MS.

**Results:** Apigenin (**1**), ferulic acid (**2**), vitexin (**3**), caprolactam (**4**), rosmarinic acid (**5**), and globoidnan A (**6**) were isolated and identified. Globoidnan A (**6**), vitexin (**3**), and rosmarinic acid (**5**) revealed the excellent DPPH^•^ scavenging effect with IC_50_ values of 22.4, 31.4, 47.2 μM, respectively. Vitexin (**3**) (IC_50_ 3.6), globoidnan A (**6**) (IC_50_ 4.6), apigenin (**1**) (IC_50_ 8.9) and ferulic acid (**2**) exhibited more ABTS^•+^ activity than standard Trolox (IC_50_ 13.8 μg/mL). Vitexin (**3**) revealed the most antiproliferative activity against HeLa, HT29, C6 and Vero cells lines with IC_50_ values of 35.6, 32.5, 41.6, 46.7 (μM), respectively.

**Discussion and conclusion:** Globoidnan A (**6**) has the most antioxidant effects on all assays. This has to do with the chemical structure of the compound bearing the acidic protons. Vitexin (**3**) could be a promising anticancer agent.

## Introduction

Plants have played a significant role in drug discovery and development process. In many nations, medicinal plants have been the main source of primary healthcare. Nearly 80% of world populations rely on folk medicine to treat various illnesses. The bioactive secondary metabolites isolated from plants have constituted the drug discovery. Recently, there has been a significant increase in the discovery of molecular targets that may be applied to the discovery of novel materials for the diagnosis and treatment of human diseases (Newman & Cragg 2007).

Due to the difficulty of synthesis, polyphenols, bioactive natural products, play an important role in the human need to satisfy their phenolic requirements through daily consumption of fruits and vegetables. Phenolic compounds are effective in the treatment of chronic and acute diseases such as cancer, cardiovascular disorders and inflammation (Baiano et al. 2014).

*Origanum* (Lamiaceae) has 23 species and six hybrids in Turkey flora, 14 of which are endemic (Baser 2002). *Origanum*, widely used in food industry and traditional medicine in many countries, is an important medicinal and aromatic plant (Wakim et al. 2013). *Origanum* species have been consumed as a folk medicine for treatment of sickness such as indigestion, headache, muscle pains, diarrhoea, rheumatism, asthma as herbal tea (Jun et al. 2001). A good deal of work has been carried out with *Origanum* on the chemical composition of essential oils which have been applied in flavouring of various foods, particularly sauces, fish, soups, meat, canned foods and bitters (Busatta et al. 2008). In addition, this genus exhibits antimicrobial activity against foodborne bacteria and fungi (El-Ashmawy et al. 2007). This plant genus exhibits biological activities such as antioxidant (Papageorgiou et al. 2008; Baatour et al. 2011), antiulcerogenic (Al-Howiriny et al. 2009), antimicrobial (Kacaniova et al. 2014), antiviral (Zhang et al. 2014), antinociceptive (Pahlavan et al., 2013), antitumour (Thoppil et al. 2013; Tuncer et al. 2013) and antifungal (Fadel et al. 2013). Previous phytochemical and pharmacological studies on the *Origanum* have revealed the presence of monoterpenes (Exarchou et al. 2013), diterpenes (Takeda et al. 2008), flavonoids (Lin et al. 2003), steroids (Bellakhdar et al. 1988), hydroquinone, hydroquinone-monomethyl ether (Assaf et al. 1987), flavone glycosides (Chatzopoulou et al. 2010) and other pharmaceutically and medicinally valuable compounds (Lin et al. 2003). Due to the biological and chemical diversities, the demand of *Origanum* in the world market has increased steadily. Turkey is the leading country for the production and exportation of *Origanum* (Ozkan et al. 2010).

Free oxygen radicals, called reactive oxygen species (ROS), comprising hydroxyl radical, superoxide and singlet oxygen, can yield cellular harm leading to diseases such as DNA damage (Pelicano et al. 2009), cancer (Lambert & Yang 2003), cardiovascular diseases (Diaz et al. 1997), Parkinson (Perfeito et al. 2012) and Alzheimer’s disease (Rottkamp et al. 2000). Even though ROS are formed by metabolic functions, they could be scavenged by compounds existing in fruits, vegetables and herbs. Hence, the extracts of fruits, vegetables and herbs containing most antioxidant compounds are important for pharmacology and food industry (Bae et al. 2012). Antioxidants, revealing significant effects for health by reduction of the oxidative stress, are used to prevent food from oxidative deterioration (Narita & Inouye 2012). Therefore, natural and synthetic antioxidants are employed to protect fresh food. Butylated hydroxytoluene (BHT), propyl gallate (PG), butylated hydroxyanisole (BHA), *tert*-butylhdroquinone (TBHQ) are commonly used as synthetic antioxidants. However, due to the toxicity of these chemicals, demand for natural antioxidants has increased steadily (Liu et al. 2011). Natural antioxidants also have diverse biological effects such as antiviral, antiinflammatory, antithrombotic, antibacterial and vasodilatory activities (Cook & Samman 1996).

Cancer is a major health problem and is the second leading cause of death worldwide. As reported by World Health Organization (WHO) assessment, by 2050, 17.5 million cancer deaths will have occurred in the world. The current chemotherapeutic drugs are highly toxic, expensive and activate the alternative cell signaling pathways. Therefore, they have limited therapeutic success in cancer (Khan et al. 2015). Cancer chemoprevention by natural products has appeared as a promising and efficient approach to curtail the cancer risk and has gained great interest since it is regarded as safe, cheap and complementary medicine to current healthcare system (Khan et al. 2016). Plants have been used in the treatment of cancer for years and have gained the importance to be most attractive source of anticancer drugs. The secondary metabolites such as alkaloids and polyphenols isolated from plants have been reported for their anticancer activities (Onrubia et al. 2013).

In this work, secondary metabolites were isolated by chromatographic techniques and structures of isolated compounds were elucidated by spectroscopic methods. Antiproliferative effects and antioxidant activity of isolated compounds are presented.

## Materials and methods

### General experimental procedures

NMR spectra were recorded on a spectrometer operated at 400 MHz for ^1 ^H and 100 MHz for ^13 ^C NMR. Chemical shifts were given in ppm (*δ* scale), coupling constant *(J)* in Hz. Column chromatography was performed on silica (60–230 mesh, Merck). TLC was carried out on analytical alumina plates (60 F_254_). Hitachi U-290 UV-Vis spectrometer was used for UV measurements. HRMS analyses were recorded on Agilent 6210 LC-TOF/MS spectrometer. Ammonium thiocyanate and BHT were purchased from E. Merck (Darmstadt, Germany). Ferrous chloride, BHA and trichloracetic acid (TCA), formic acid, foetal bovine serum (FBS), penicillin/streptomycin and Dulbecco’s modified Eagle’s medium-high glucose (DMEM-HG) were purchased from Sigma-Aldrich (Darmstadt, Germany).

### Plant material

The plant materials were collected from Artvin, east part of Turkey during the flowering period in July 2014 and identified by Prof. Dr Ozgur Eminagaoglu, specialists of Plant Taxonomy at Department of Forestry Engineering, Faculty of Forestry, Artvin Coruh University where a voucher specimen was deposited (No: ARTH 5247). The *O. rotundifolium* was dried in shade, finely powdered for extraction process.

### Extraction and isolation

*Origanum rotundifolium* aerial part was dried and powdered (1.0 kg ±2.0 g) then extracted with hexane (3 × 1 L) for a week at room temperature, filtered and solvent was removed to obtain hexane extract. Solid material of hexane extract was re-extracted with ethyl acetate (3 × 1 L) for a week at room temperature, filtered and after removal of the solvent, ethyl acetate extract was achieved. Methanol extract was acquired by extracting (3 × 1 L) the solid part of ethyl acetate extract for a week at room temperature. The solid part of methanol extract was heated with distillated water (500 mL) at 80 °C for 2 h, filtered then 100 mL of which was lyophilized to get the water extract. The other part of the water extract (400 mL) was partitioned with ethyl acetate, and ethyl acetate phase was separated by separator funnel, dried with (MgSO_4_), filtered then solvent was removed to obtain the water/ethyl acetate extract revealing the most antioxidant activity and including the highest phenolic contents. Therefore, chromatographic techniques were applied for water/EtOAc extract to isolate the bioactive compounds. The water/EtOAc extract (14 g) was subjected to silica gel column chromatography (100 × 2.5 cm), eluted with a solvent system with increasing polarity from hexane to EtOAc and EtOAc-MeOH. 400 fractions, each 100 ml were collected. After checking the thin-layer chromatography, the compounds having same *R*_f_ values were combined. Apigenin (**1**) was isolated from the fractions of 154–182 with further purification of silica gel column chromatography. The fractions 183–188 included the ferulic acid (**2**). 190–220 fractions were combined and chromatographed on silica gel to isolate vitexin (**3**). Caprolactam (**4**) was isolated as pure form from fractions 230–260. Rosmarinic acid (**5**) was isolated from the fractions 300–330. The fractions 340–400 were combined and subjected to column chromatography to isolate the globoidnan A (**6**).

### Antioxidant assays

#### Determination of total phenolic compounds

Gallic acid and Folin–Ciocalteu reagent were used to determine the total phenolic constituent of extract (Slinkard & Singleton 1977). Stock solution was prepared by dissolving the extract (1.0 mg) in MeOH (1.0 mL). An aliquot of stock solution (0.1 mL) was added to test tube then deionized water (4.5 mL), a solution of Folin-Ciocalteu reagent (0.1 mL) and Na_2_CO_3_ (0.3 mL, 2%) were added. After stirring 3 min vigorously, the mixture was kept for 2 h incubation. The absorbance measurement was carried out at 760 nm in a spectrophotometer. The total phenolic constituents concentrations in the extracts were calculated as gram of gallic acid equivalent by using the below equation.

Absorbance=1.018×Total phenols [Gallic Acid Equivalent(mg)]−0.001

#### DPPH^•^ scavenging assay

The free radical scavenging activities of extracts and standards were evaluated by 2,2-diphenyl-1-picrylhydrazil (DPPH^•^) (Blois 1958). To a different concentration of 3.0 mL of extract (3.0–20 μg/mL) in ethanol, DPPH^•^ solution (1.0 mL, 0.26 mM) were added. After that it is stirred and kept at *R*t for 30 min, The absorbance of the mixture was executed at 517 nm in a spectrophotometer. The activity was calculated by the below equation.

$$DPPH\text{•}\;scavenging\;effect\;\left(\%\right)=\left[\frac{Ac-As}{Ac}\right]\times 100$$

In which, *A*c and *A*s are the control and sample absorbance, respectively.

#### ABTS^•+^ scavenging assay

2,2″-Azino-bis (3-ethylbenzthiazoline-6-sulfonic acid) (ABTS) cation radical scavenging assay based on the decreasing ABTS radical cation, a blue/green chromophore with absorption at 737 nm, in comparison to that of BHA, BHT and Trolox. This method was executed as in the literature (Re et al. 1999). Treatment of ABTS (2.0 mM) in phosphate buffer (0.1 M, pH 7.4) with potassium persulfate (K_2_S_2_O_8_) (2.45 mM) gave the ABTS^•+^, kept at dark at *R*t for 4 h. Initially, dilution of ABTS^•+^ was performed with sodium phosphate buffer (pH 7.4, 0.1 M) to obtain absorbance 0.750 ± 0.025 at 734 nm. After all, ABTS^•+^ solution (1.0 mL) was added to each extract solution in ethanol (3.0 mL) at different concentration (2.5–20 μg/mL). After 30 min, the inhibition was calculated for each concentration relative to a blank absorbance. The scavenging ability of ABTS^•+^ was calculated by given equation:

${\text{ABTS}}^{•+}\text{scavenging activity }\left(\%\right)=\left[\left(\text{Ac }–\text{ As}\right)/\text{Ac}\right]*\text{1}00$ in which, *A*c is the initial concentration of ABTS^•+^ and *A*s is the absorbance of the remaining concentration of ABTS^•+^ in the samples.

#### Ferric ions (Fe^3+^) reducing antioxidant power assay (FRAP)

Different concentrations of samples (5–40 μg/mL) in 1 mL of deionized water were mixed with sodium phosphate buffer (1.25 mL, 0.2 M, pH 6.6) and potassium ferricyanide [K_3_Fe(CN)_6_] (1.25 mL, 1%). After the incubation of the mixture at 50 °C for 20 min, trichloroacetic acid (1.25 mL, 10%) and FeCl_3_ (0.25 mL, 0.1%) were added to the reaction medium. The reaction mixture was vortexed thoroughly and absorbance measurement at 700 nm was executed in a spectrophotometer. Increase in the absorbance reveals the increase in the reduction capability of the reaction mixture (Oyaizu 1986).

### Antiproliferative assays

#### Preparation of cell culture

Anticancer potential of isolated compounds was investigated on C6, HT29, HeLa and Vero cell lines. These cells were sustained with Dulbecco’s modified eagle’s medium, complemented with foetal bovine serum and PenStep solution (ATCC, American Type Culture Collection). The old medium was aspirated out of the plate while cells had reached a confluence of 80% then cells were removed from the flasks using trypsin-EDTA (4 mL) and centrifuged. Afterward, cell pellet was suspended with DMEM and was counted to gain a final concentration as 5 × 10^4^ cells/mL, inoculated into wells (100 μL cells/well).

#### Cell proliferation assay

The antiproliferative activity of compounds against various cell lines (C6, HeLa and Vero cells) was tested by cell proliferation assay using BrdU Cell Proliferation ELISA kit. A cell suspension containing nearly 5 × 10^3^ cells in 100 μL was conveyed into the well cell plates (96-well cell). The treatments of cells with compounds in DMSO and 5-Florouracil (5 FU) in DMSO separately at concentration 5–100 μg/mL were carried out. The last volume of the wells was adapted to 200 μL by DMEM and mixture was incubated at 37 °C with 5% CO_2_ for overnight (Okten et al. 2013).

#### IC_50_ and % inhibition

The half inhibitory concentration of the isolated compounds and standards was calculated by XLfit5 software (IDBS) and explicated in μM at 95% confidence levels (Table 1). The proliferation results were recorded as the per cent inhibition of the compounds and control substances. The following formula was used for the calculation of inhibition

$$\%\text{ Inhibition }=\;\left[A_{\text{sample}}–\;A_{\text{control}}/A_{\text{control}}\right]\times \text{1}00$$

**Table 1.** IC_50_ values and tumour specificity rate for compounds.

	IC_50_ (μM)				Tumour specificity		
Compounds	HeLa	HT29	C6	Vero	HeLa	HT29	C6
1	ND	ND	ND	ND	–	–	–
2	402.63	411.73	314.19	472.77	1.17	1.15	1.50
3	35.57	32.49	41.60	46.70	1.31	1.43	1.12
4	ND	ND	ND	ND	–	–	–
5	232.80	261.85	251.47	279.35	1.19	1.07	1.11
6	140.07	136.33	169.01	ND	–	–	–
Cisplatin	44.25	48.58	61.45	40.78	0.92	0.84	0.66

ND: not determined.

where *A*_sample_ is the absorbance of the compounds and *A*_control_ is the absorbance of the DMSO.

#### Cytotoxicity assay

The cytotoxicity of isolated compounds and standard on C6, HT29, HeLa and Vero cells was presented through a Lactate Dehydrogenase (LDH) Cytotoxicity Detection Kit (Roche, USA). Almost 5 × 10^3^ cells in 100 μL were seeded into 96-well microtiter plates and treated with 5, 10, 20, 30, 40, 50, 75 and 100 μg/mL concentrations of these compounds at 37 °C with 5% CO_2_ overnight. LDH activity was executed by evaluating the absorbance at 492–630 nm by a microplate reader.

### Statistical analysis

Statistical analyses were performed using the Statistical Package for Social Sciences (Windows version 21.0: SPSS). Data were reported as mean ± standard deviation. Between-group statistical differences for parametric data were analyzed using the Student’s *t* test or Duncan’s Multiple-Range Test was used. *p* < 0.05 was considered as significant for all the tests.

## Results and discussion

### Natural products

**Figure 1.**
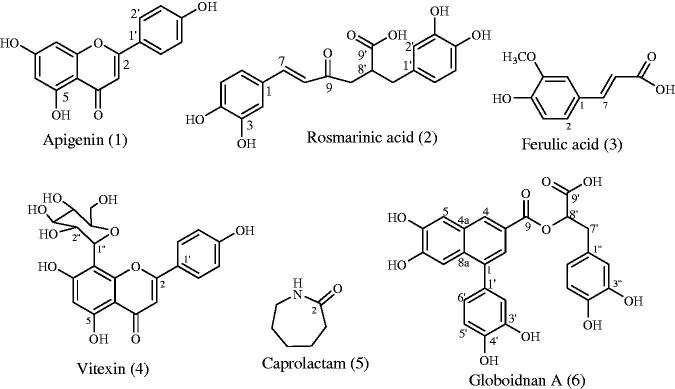
Isolated compounds from *O. rotundifolium*.

After a series of column chromatography, apigenin (**1**) was isolated as white powder. The ^13 ^C NMR spectrum and also DEPT experiments showed 15 signals containing seven methines, seven quaternary carbon atoms and one carbonyl carbon that complied with the structure. In ^1 ^H NMR spectrum, observation of signals at *δ* 7.95 (d, *J* = 8.8 Hz, H6′), *δ* 7.93 (d, *J* = 8.8 Hz, H2′), *δ* 6.94 (d, *J* = 8.8 Hz, H3′, H5′), *δ* 6.50 (d, *J* = 1.8 Hz, H8), *δ* 6.18 (d, *J* = 1.8 Hz, H6) accorded with the compound (Owen et al. 2003). Ferulic acid (**2**) was isolated as white solid. The ^13 ^C spectrum revealing the presence of one methoxy, five methines, three quaternary carbons and one carbonyl carbon suited with the structure. In ^1 ^H NMR spectrum, the signal observed at *δ* 3.81 as singlet belonged to methoxy group. The double bond protons’ signals at *δ* 7.46 (H8) and *δ* 6.36 (H7) with large coupling constant (*J* = 16.0) revealed the linkage of acrylic acid to the phenyl moiety with trans manner. The signals appeared at *δ* 7.27 as a broad singlet belonged to the H6. H2 and H3 proton resonated at *δ* 7.06 and 6.78 with 8.0 coupling constant, respectively (Durust et al. 2001). Vitexin (**3**) (Wen et al. 2007), caprolactam (**4**) (Dahlhoff et al. 2001), rosmarinic acid (**5**) (Erenler et al. 2016a), globoidnan A (**6**) (Erenler et al. 2016b) were isolated successively (Figure 1). Their structures were elucidated by spectroscopic techniques basically 1 D NMR, 2 D NMR, LC-TOF/MS and comparison of which with those reported in the literatures. All spectra of isolated compounds were given as Supplementary material.

### Total phenolic contents

In this work, effective extraction method was developed by a series of extraction techniques to yield the strong antioxidant activities. Interestingly, the total phenolic content of water/EtOAc extract consists of four times as much as phenolic compounds than the methanol extract which is the second one. This also led to the best antioxidant activities. The order of total phenolic content is as follows: gallic acid/kg extract, water/EtOAc extract (620.5) > MeOH extract (164.7) > water extract (134.6) > EtOAc extract (44.9) > hexane extract (21.9).

### DPPH free radical scavenging activity

**Figure 2.**
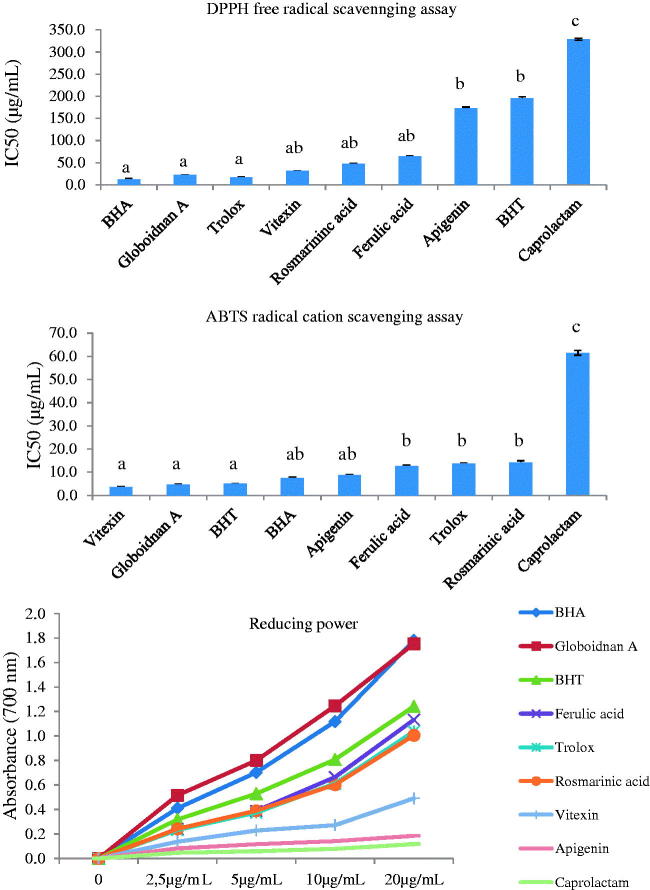
DPPH^•^ scavenging, ABTS^•+^ scavenging and reducing power activities of isolated compounds and positive controls. (The same letters on the columns of DPPH and ABTS revealed that the differences are not significant statistically (*p* > 0.05). The different letters indicated that the differences are significant (*p* < 0.05).

The mechanism of DPPH^•^ scavenging activity is based on the hydrogen or electron releasing capability of antioxidant molecules to DPPH^•^ molecules to form the nonradical DPPH-H and the measurement of the reducing ability of antioxidants. When DPPH radicals react with a proton donor molecule like an antioxidant, the radicals are scavenged and the absorbance is reduced. During the reduction process, purple DPPH^•^ changes to a colourless diphenyl picrylhydrazine and remaining DPPH^•^ exhibiting the maximum absorption at 517 nm is measured (Gulcin 2012). In this work, water/EtOAc extract exhibited the best DPPH^•^ scavenging activity. Therefore, bioactive compounds were isolated from this extract. The isolated compounds bearing acidic protons revealed the excellent antioxidant activity. The order of DPPH free radical scavenging activity is as follows (μM): BHA (IC_50_ 12.9) > globoidnan A (**6**) (IC_50_ 22.4) > Trolox (IC_50_ 17.3) > vitexin (**3**) (IC_50_ 31.4) > rosmarinic acid (**5**) (IC_50_ 47.2) > ferulic acid (**2**) (IC_50_ 64.3) > apigenin (**1**) (IC_50_ 173.4) > BHT (IC_50_ 196.1) > caprolactam (**4**) (IC_50_ 328.8) (Figure 2). Globoidnan A (**6**) has carboxylic acid group, hence it can donate the proton to the DPPH radical easily and bearing the hydroxyl groups of Globoidnan A (**6**) also facilitates the proton donation. Therefore, this compound revealed the excellent DPPH^•^ scavenging activity.

### ABTS radical cation decolourization assay

In this assay, ABTS is oxidized to its radical cation form, ABTS˙^+^ which is blue–green coloured. The antioxidant ability of extracts and compounds is measured by detecting the decreasing colour due to the reaction of extracts with ABTS˙^+^. Water/EtOAc extract revealed the most ABTS radical cation activity among the extracts. The order of activity of isolated compounds is as follows: vitexin (**3**) (IC_50_ 3.7) > globoidnan A (**6**) (IC_50_ 4.6) > BHT (IC_50_ 5.0) > BHA (IC_50_ 7.6) > apigenin (**1**) (IC_50_ 8.9) > ferulic acid (**2**) (IC_50_ 12.6) > Trolox (IC_50_ 13.8) > rosmarinic acid (**5**) (IC_50_ 14.2) > caprolactam (**4**) (IC_50_ 61.5) (Figure 2). Vitexin (**3**) activity is close to globoidnan A one. The active compounds bear protons to give to the ABTS˙^+^. This spectrophotometric technique is easy and simple for screening and routine analyses. This method is based on the inhibition of ABTS˙^+^ by abstracting proton or electron from antioxidants. ABTS˙^+^ is more reactive than DPPH^•^. ABTS radical scavenging method is applicable in a wide pH range that is essential for food analysis (Gulcin 2012).

### Reducing power

Reduction of Fe^+3^ to Fe^+2^ is determined by measuring the absorbance of Perl’s Prussian blue complex (Gulcin 2009). The reduction capacity of extract exhibits that it has a significant antioxidant potential. Antioxidant compounds in extracts are able to donate electrons to radicals which are reduced to more stable and unreactive species. The reducing power of extracts was investigated by Fe^+3^ to Fe^+2^ transformation assay. Ferric reduction powers of isolated compounds were given in Figure 2 and results were compared to BHT, BHA and Trolox. Globodnin A had higher activity than BHT, BHA and Trolox. The differences were statistically significant (*p* < 0.05). The reducing power of isolated compounds decreases in the following order (10 μg/mL): globoidnan A (**6**) (1.25) > BHA (1.12) > BHT (0.81) > ferulic acid (**2**) (0.67) > Trolox (0.61) > rosmarinic acid (**5**) (0.60) > vitexin (**3**) (0.27) > apigenin (**1**) (0.14) > caprolactam (**4**) (0.08).

### Activity of compounds against C6 (rat brain tumour) cell lines

**Figure 3.**
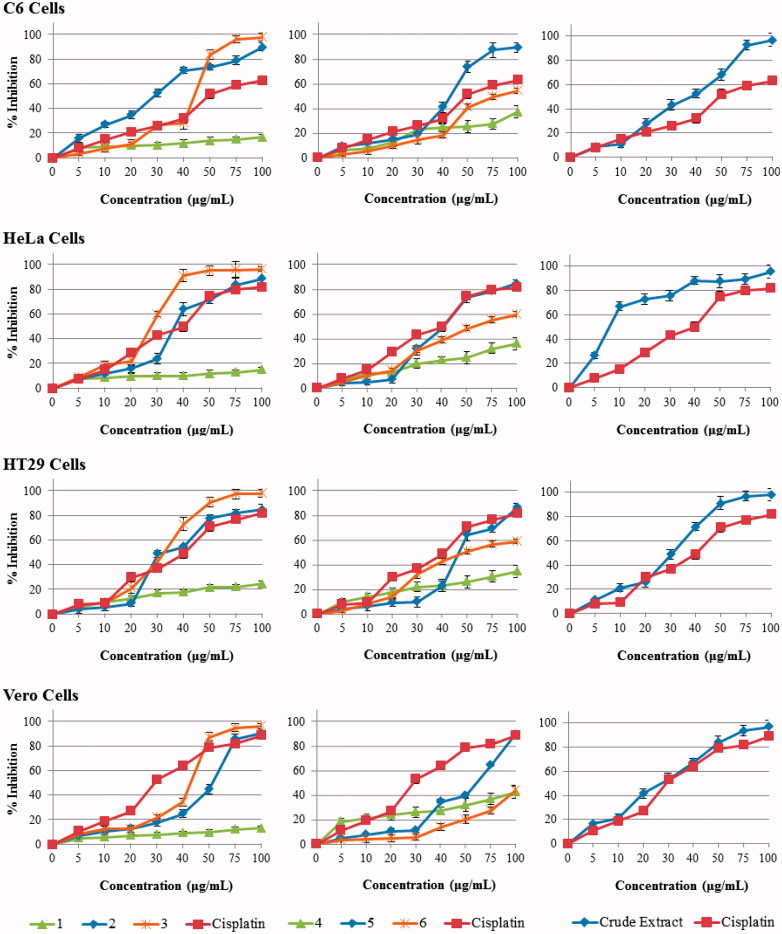
The antiproliferative activity of compounds on C6, HeLa, HT29 and Vero cell lines. Cell proliferation was measured using a BrdU Cell Elisa Assay Kit. Percent inhibition was reported as mean values ± SEM of three independent assays (*p* < 0.05). Each experiment was repeated three times for each cell line.

Vitexin (**3**) revealed the highest activity on C6 cells lines (μM, IC_50_, 41.6) than the tested compounds including cisplatin (IC_50_, 61.5). Rosmarinic acid (**2**) and globoidnan A exhibited moderate activity with IC_50_ values of 251.5 and 169.0 μM, respectively. Noticeable effect has not been detected for apigenin (**1**) and caprolactam (**4**) on C6 cells lines. The extract activity was better than the standard. This may be either the synergic effect of compounds in the extract or consisting of active compound (Figure 3).

### Activity of compounds and extract against HeLa (human cervix carcinoma) cell lines

The isolated compounds and extract was evaluated against HeLa cell lines at various concentrations. Apigenin (**1**) did not reveal the noticeable activity whereas, vitexin (**3**) (IC_50_, 35.6) and ferulic acid (**2**) (IC_50_, 40.6), showed the higher activity than the standard (IC_50_, 44.3). Rosmarinic acid (**5**) (IC_50_, 232.8), globoidnan A (**6**) (IC_50_, 140.1) displayed more or less activity than the 5-Florourasil at concentration dependence (Figure 3). The outstanding activity of extract may be due to the synergic effect of compounds into the extract.

### Activity of compounds and extract against HT29 (human colon carcinoma) cell lines

Apigenin (**1**) has not a noteworthy effect but vitexin (**3**) (IC_50_, 32.5) has excellent effect on HT29 cell lines. Rosmarinic acid (**5**) (IC_50_, 261.9) revealed the slightly activity between the concentrations of 5–30 μg/mL but after this concentration the activity increased sharply. While globoidnan A (**6**) (IC_50_, 136.3) showed the good activity, the extract exhibited excellent activity (Figure 3).

### Activity of compounds and extract against vero (African green monkey kidney epithelium) cells lines

The activity tests of isolated compounds and extract were executed on Vero cell lines as well and similar trend was observed on this cell lines. Ferulic acid (**2**) and vitexin (**3**) were effective at high concentrations. Rosmarinic acid (**5**) and extract have good effect on this cell lines but apigenin (**1**) caprolactam (**4**) globoidnan A (**6**) did not reveal the remarkable activity (Figure 3). The isolated compounds did not reveal the significant toxicity on all cell lines.

## Conclusions

*Origanum rotundifolium* is an important aromatic and medicinal plant including significant bioactive compounds. Apigenin (**1**), ferulic acid (**2**), vitexin (**3**), caprolactam (**4**), rosmarinic acid (**5**), globoidnan A (**6**) were isolated from this plant by chromatographic methods and structures of these compounds were elucidated by spectroscopic techniques. These compounds exhibited the excellent antioxidant activities. Moreover, ferulic acid (**2**), vitexin (**3**), rosmarinic acid (**5**), globoidnan A (**6**) and extract revealed the good antiproliferative activity on C6 (rat brain tumour), HeLa (human cervix carcinoma), HT29 (human colon carcinoma) and Vero (African green monkey kidney epithelium) cells lines. The isolated compounds and extract of *O. rotundifolium* has the potency to be used in food industries as a natural antioxidant as well as pharmaceutical trades. These compounds were isolated from *O. rotundifolium* for the first time.

## Supplementary Material

Ramazan_Erenler_et_al_supplemental_content.zip
