# Supplementary material for: Bioassay-guided isolation, identification of compounds from Origanum rotundifolium and investigation of their antiproliferative and antioxidant activities
Source: Pharm Biol. 2017 Apr 21;55(1):1646–53. doi: 10.1080/13880209.2017.1310906 (PMC7012012; doi:10.1080/13880209.2017.1310906)
Supplement: Ramazan_Erenler_et_al_supplemental_content.zip [file IPHB_A_1310906_SM2031.zip › Ramazan Erenler et al supplemental content.doc]

**SUPPORTING MATERIALS**

**Bioassay guided isolation, identification of compounds from *Origanum*** ***rotundifolium* Boiss. and investigation of their antiproliferative and antioxidant activities**

Ramazan Erenler^[1]^, Bilal Meral^1^, Ozkan Sen^1^, Mahfuz Elmastas^1^, Ali Aydin^2^, Ozgur Eminagaoglu^3^, Gulacti Topcu^4^

*^1^Department of Chemistry, Faculty of Art and Science, Gaziosmanpasa University, 60240 Tokat, Turkey,* *^2^Department of Biology, Faculty of Art and Science, Gaziosmanpasa University, 60240 Tokat, Turkey, ^2^Department of Forest Engineering, Faculty of Foresty, Artvin Coruh University, 08000 Artvin, Turkey, ^3^Department of Pharmacognosy and Phytochemistry, Faculty of Pharmacy, Bezmialem Vakif University, 34093 Istanbul, Turkey*

**Table of Contents	Page**

**S1:** ^1^H-NMR (400 MHz, DMSO-*d6*) Spectrum of Compound **1** (apigenin)	3

**S2:** ^13^C-NMR (100 MHz, DMSO-*d6*) Spectrum of Compound **1** (apigenin)	4

**S3:** HETCOR (400 MHz, DMSO-*d6*) Spectrum of compound **1 (**apigenin)	5

**S4:** HMBC (400 MHz, DMSO-*d6*) Spectrum of compound **1 (**Apigenin)	6

**S5:** COSY-90 (400 MHz, DMSO-*d6*) Spectrum of compound **1** (apigenin)	7

**S6:** HPLC-QTOF Spectrum of compound **1** (apigenin)	8

**S7:** ^1^H-NMR (400 MHz, DMSO-*d6*) Spectrum of Compound **2** (rosmarinic acid)	9

**S8:** ^13^C-NMR (100 MHz, DMSO-*d6*) Spectrum of Compound **2** (rosmarinic acid)	10

**S9:** DEPT-135, APT, DEPT-90 NMR (100 MHz, DMSO-*d6*) Spectrum of

compound **2** (rosmarinic acid)	11

**S10:** ^1^H-NMR (400 MHz, DMSO-*d6*) Spectrum of Compound **3** (ferulic acid)	12

**S11:** ^1^H-NMR (400 MHz, DMSO-*d6*) Spectrum of Compound **4** (vitexin)	13

**S12:** ^13^C-NMR (100 MHz, DMSO-*d6*) Spectrum of Compound **4** (vitexin)	14

**S13:** DEPT-135, APT, DEPT-90 NMR (100 MHz, DMSO-*d6*) Spectrum of

compound **4** (vitexin)	15

**S14:** ^1^H-NMR (400 MHz, DMSO-*d6*) Spectrum of Compound **5** (caprolactam)	16

**S15:** ^13^C-NMR (100 MHz, DMSO-*d6*) Sp ectrum of Compound **5** (caprolactam)	17

**S16:** ^1^H-NMR (400 MHz, DMSO-*d6*) Spectrum of Compound **6** (globoidnan A)	18

**S17:** ^13^C-NMR (100 MHz, DMSO-*d6*) Spectrum of Compound **6** (globoidnan A)	19

**S18:** DEPT-90 NMR (100 MHz, DMSO-*d6*) Spectrum of compound **6 (**globoidnan A)	20

**S19:** HETCOR (400 MHz, DMSO-*d6*) Spectrum of compound **6 (**globoidnan A)	21

**S20:** HMBC (400 MHz, DMSO-*d6*) Spectrum of compound **6 (**globoidnan A)	22

**S21:** COSY-90 (400 MHz, DMSO-*d6*) Spectrum of compound **6 (**globoidnan A)	23

**S1:** ^1^H-NMR (400 MHz, DMSO-*d6*) Spectrum of Compound **1** (apigenin)

**S2:** ^13^C-NMR (100 MHz, DMSO-*d6*) Spectrum of Compound **1** (apigenin)

**S3:** HETCOR (400 MHz, DMSO-*d6*) Spectrum of compound **1 (**apigenin)

**S4:** HMBC (400 MHz, DMSO-*d6*) Spectrum of compound **1 (**Apigenin)

**S5:** COSY-90 (400 MHz, DMSO-*d6*) Spectrum of compound **1** (apigenin)

**S6:** HPLC-QTOF Spectrum of compound **1** (apigenin)

**S7:** ^1^H-NMR (400 MHz, DMSO-*d6*) Spectrum of Compound **2** (rosmarinic acid)

**S8:** ^13^C-NMR (100 MHz, DMSO-*d6*) Spectrum of Compound **2** (rosmarinic acid)

**S9:** DEPT-135, APT, DEPT-90 NMR (100 MHz, DMSO-*d6*) Spectrum of compound **2** (rosmarinic acid)

**S10:** ^1^H-NMR (400 MHz, DMSO-*d6*) Spectrum of Compound **3** (ferulic acid)

**S11:** ^1^H-NMR (400 MHz, DMSO-*d6*) Spectrum of Compound **4** (vitexin)

**S12:** ^13^C-NMR (100 MHz, DMSO-*d6*) Spectrum of Compound **4** (vitexin)

**S13:** DEPT-135, APT, DEPT-90 NMR (100 MHz, DMSO-*d6*) Spectrum of compound **4** (vitexin)

**S14:** ^1^H-NMR (400 MHz, DMSO-*d6*) Spectrum of Compound **5** (caprolactam)

**S15:** ^13^C-NMR (100 MHz, DMSO-*d6*) Spectrum of Compound **5** (caprolactam)

**S16:** ^1^H-NMR (400 MHz, DMSO-*d6*) Spectrum of Compound **6** (globoidnan A)

**S17:** ^13^C-NMR (100 MHz, DMSO-*d6*) Spectrum of Compound **6** (globoidnan A)

**S18:**, DEPT-90 NMR (100 MHz, DMSO-*d6*) Spectrum of compound **6 (**globoidnan A)

**S19:** HETCOR (400 MHz, DMSO-*d6*) Spectrum of compound **6 (**globoidnan A)

**S20:** HMBC (400 MHz, DMSO-*d6*) Spectrum of compound **6 (**globoidnan A)

**S21:** COSY-90 (400 MHz, DMSO-*d6*) Spectrum of compound **6 (**globoidnan A)

1. Correspondence: Professor Ramazan Erenler, Department of Chemistry, Faculty of Art and Science, Gaziosmanpasa University, 60240 Tokat, Turkey; E-mail: [ramazan.erenler@gop.edu.tr](mailto:ramazan.erenler@gop.edu.tr); Tel.+903562521616-3055; Fax:+903562521585
